# *Plasmodium* microtubule-binding protein EB1 is critical for partitioning of nuclei in male gametogenesis

**DOI:** 10.1128/mbio.00822-23

**Published:** 2023-08-03

**Authors:** Sydney Mauer, Nelly Camargo, Biley A. Abatiyow, Olivia R. Gargaro, Stefan H. I. Kappe, Sudhir Kumar

**Affiliations:** 1 Center for Global Infectious Disease Research, Seattle Children’s Research Institute, Seattle, Washington, USA; 2 Department of Global Health, University of Washington, Seattle, Washington, USA; 3 Department of Pediatrics, University of Washington, Seattle, Washington, USA; Albert Einstein College of Medicine, Bronx, New York, USA

**Keywords:** *Plasmodium*, microtubules, gamete, nucleus, mosquito, transmission

## Abstract

Sexual reproduction of the malaria parasites is critical for their transmission to a mosquito vector. Several signaling molecules, such as kinases and phosphatases, are known to regulate this process. We previously demonstrated that *Plasmodium falciparum* (*Pf*) Ca^2+^-dependent protein kinase 4 (CDPK4) and serine/arginine-rich protein kinase 1 (SRPK1) are critical for axoneme formation during male gametogenesis, with genetic deletion of either gene causing a complete block in parasite transmission to the mosquito. A comparative phospho-proteome analysis of *Pfcdpk4^−^* and RNA-seq analysis of *Pfsrpk1^−^* gametocytes showed that these kinases regulate similar biological processes linked to both microtubule (MT) dynamics and cell motility. One of these proteins was a nuclear MT-associated End Binding protein 1 (EB1), which was hypophosphorylated in *Pfcdpk4*^−^ gametocytes. To study the functional relevance of EB1, we created gene deletion parasites for *EB1*. We further demonstrate that *Pfeb1*^−^ parasites like WT NF54 parasites proliferate normally as asexuals and undergo gametocytogenesis and gametogenesis. Strikingly, these parasites suffer a severe defect in nuclear segregation and partitioning of nuclei into emerging microgametes. Further genetic crosses utilizing male- and female-sterile parasites revealed that *Pfeb1^−^* parasites only suffer a male fertility defect. Overall, our study reveals an essential function for *Pf*EB1 in male gamete nuclear segregation and suggests a potential therapeutic avenue in the design of transmission-blocking drugs to prevent malaria transmission from humans to mosquito.

**IMPORTANCE:**

Gametogenesis and subsequent gamete fusion are central to successful transmission of the malaria parasites to a female *Anopheles* mosquito vector and completion of the sexual phase of the parasite life cycle. Male gametogenesis involves the formation of axonemes inside male gametes from male gametocytes via active cytoskeleton remodeling. The tubulin and tubulin-binding proteins are, thus, attractive anti-malarial drug targets. In the present study, we demonstrate that a microtubule-binding protein *Pf*EB1 is essential for male gamete fertility, specifically for the inheritance of nuclei from activated male gametocytes. Targeting *Pf*EB1 function may provide new avenues into designing interventions to prevent malaria transmission and disease spread.

## INTRODUCTION

*Plasmodium* parasites utilize both vertebrate hosts and female *Anopheles* mosquito vectors for the completion of their life cycle. Sporozoites are transmitted by mosquito bite which infect hepatocytes and replicate asexually to form and release merozoites, which infect red blood cells (RBCs). Blood-stage parasites replicate asexually within RBCs, but some of these parasites commit to sexual-stage differentiation called gametocytogenesis, which proceeds through five distinct stages (stages I–V), and in the human malaria parasite *Plasmodium falciparum*, takes up to 14 days. Stage V gametocytes rapidly undergo gametogenesis when ingested by a mosquito during a blood meal. Mosquitoes are infected only by the parasite stages that arise after fertilization, and thus, malaria transmission is dependent on successful gamete development.

The morphologically distinct changes during gametocytogenesis require active cytoskeletal remodeling, and male gametes depend on microtubule (MT)-driven motility to fertilize females. MTs are cytoskeletal filaments with a diameter of ~25 nm, composed of alternating rings of α- and β-tubulin dimers (1). *Plasmodium* tubulin is more similar to that of plants than to mammalian tubulin, and inhibitors targeting parasite tubulin specifically disrupt their growth without affecting human MTs (2). More recently, antibodies targeting *Pf* α-tubulin I have shown strong transmission reducing efficacy (3). Therefore, cytoskeletal proteins such as MTs and their associated proteins (MAPs) are attractive drug or vaccine targets, especially during sexual-stage development. Tubulin-binding proteins in eukaryotic cells facilitate the assembly and disassembly of MTs, which drive cell division, differentiation, and motility (1, 4, 5). In eukaryotes, MTs also form the scaffolds of the mitotic and meiotic spindles of dividing cells (6). *Plasmodium* invasive stages and gametocytes maintain cell shape and rigidity with a pellicle undergirding the plasma membrane, which is composed of a network of subpellicular microtubules (SPMTs) (7) and associated proteins (8) underneath a double membrane known as the inner membrane complex (9, 10). Two additional populations of microtubules are found in the gametocyte nuclear spindle or hemi-spindle (11) and in the cytoplasm. While the cytoplasmic microtubules are short lived and only appear in early-stage gametocytes on the opposite side of the cell from the developing pellicle (10), the nuclear microtubules are present in stages III/IV gametocyte (7, 12).

Male gametogenesis is a rapid process and culminates in the formation of eight flagellated microgametes that egress from the infected RBC (exflagellation) (13). Microtubule-binding proteins impact flagellar formation and length via involvement in the equilibrium of assembly and disassembly of tubulin at the plus end (14, 15). In Apicomplexan parasites, the MT-binding apicortin protein complex comprise of a doublecortin domain and a partial p25α domain, which exhibit MAP-like functions (16). In rodent malaria parasite, *Plasmodium yoelii* (*Py*) (*Py*p25α) is crucial for exflagellation and, thus, essential for microgametogenesis (17). A recent study reported the nucleation of SPMTs in *P. falciparum* gametocytes at the outer centriolar plaque, a non-mitotic microtubule organizing center (MTOC) embedded in the nuclear membrane of the parasite (12). This study also reported that classical mitotic machinery components, including centriolar plaque proteins, *Pf*centrin-1 and -4, microtubule-associated protein, end-binding protein-1 (EB1), a kinetochore protein, *Pf*NDC80, and centromere-associated protein, *Pf*CENH3, are involved in the nuclear microtubule assembly/disassembly (12).

End-binding EB1 proteins are microtubule plus-end-tracking proteins (+TIPs) (18), which play a crucial role in regulating MT dynamics by binding growing MT ends and interacting with a network of +TIPs (18). In budding yeast mutants, BIM1 (an EB1 homolog) plays a role in MT search and capture, cell polarization, and chromosome stability (19). BIM1 gene deletion results in increased net polymerization (19). EB1 demonstrates a critical role in mitosis by aiding in kinetochore clustering and MT stability during segregation of chromosomes toward cell poles (20). However, in plant cells, EB1 colocalizes with MTs, demonstrates +TIP abilities, and influences MT dynamics by impacting polymerization rates (21). In the Apicomplexan parasite, *Toxoplasma gondii* (*Tg*), *Tg*EB1 is localized to the nucleus and tightly regulates MT dynamics (22). EB1 has recently been localized to the full length of the nuclear MT bundles of *P. falciparum* during asexual and sexual erythrocytic stages (12), suggesting a possible role in chromosomal segregation during these developmental stages.

We recently demonstrated that both *Pfcdpk4^−^* parasites (23) and *Pfsrpk1^−^* parasites (24) exhibit severe defects in exflagellation and microgametogenesis. Also, our transcriptomic and proteomic studies on both these parasite gene knockout lines revealed that they have a strong dysregulation in the expression of genes encoding transcripts for biological processes linked to MT and cell motility (23, 24). Interestingly, *Pf*EB1 was hypo-phosphorylated in *Pfcdpk4^−^* parasites at S^15^ (23), and its transcripts were severely downregulated in *Pfsrpk1^−^* parasites (24). Therefore, we sought to determine *Pf*EB1 function during sexual stages. We created gene deletion parasites (*Pfeb1^−^*) via CRISPR/Cas9-mediated transgenesis. *Pfeb1^−^* parasites showed normal asexual blood-stage growth and underwent normal gametocytogenesis. Strikingly, *Pfeb1^−^* parasites exhibited a robust block in transmission to mosquitoes. Careful examination of microgametes revealed a severe defect in partitioning of nuclei in microgametes in *Pfeb1^−^* parasites, while macrogametes formed normally. Further genetic crosses involving male-only and female-only sterile parasite lines demonstrated that *Pf*EB1 is critical for male gamete fertility only.

## RESULTS

### *Pf*EB1 is not required for intra-erythrocytic parasite development

**Fig 1:**
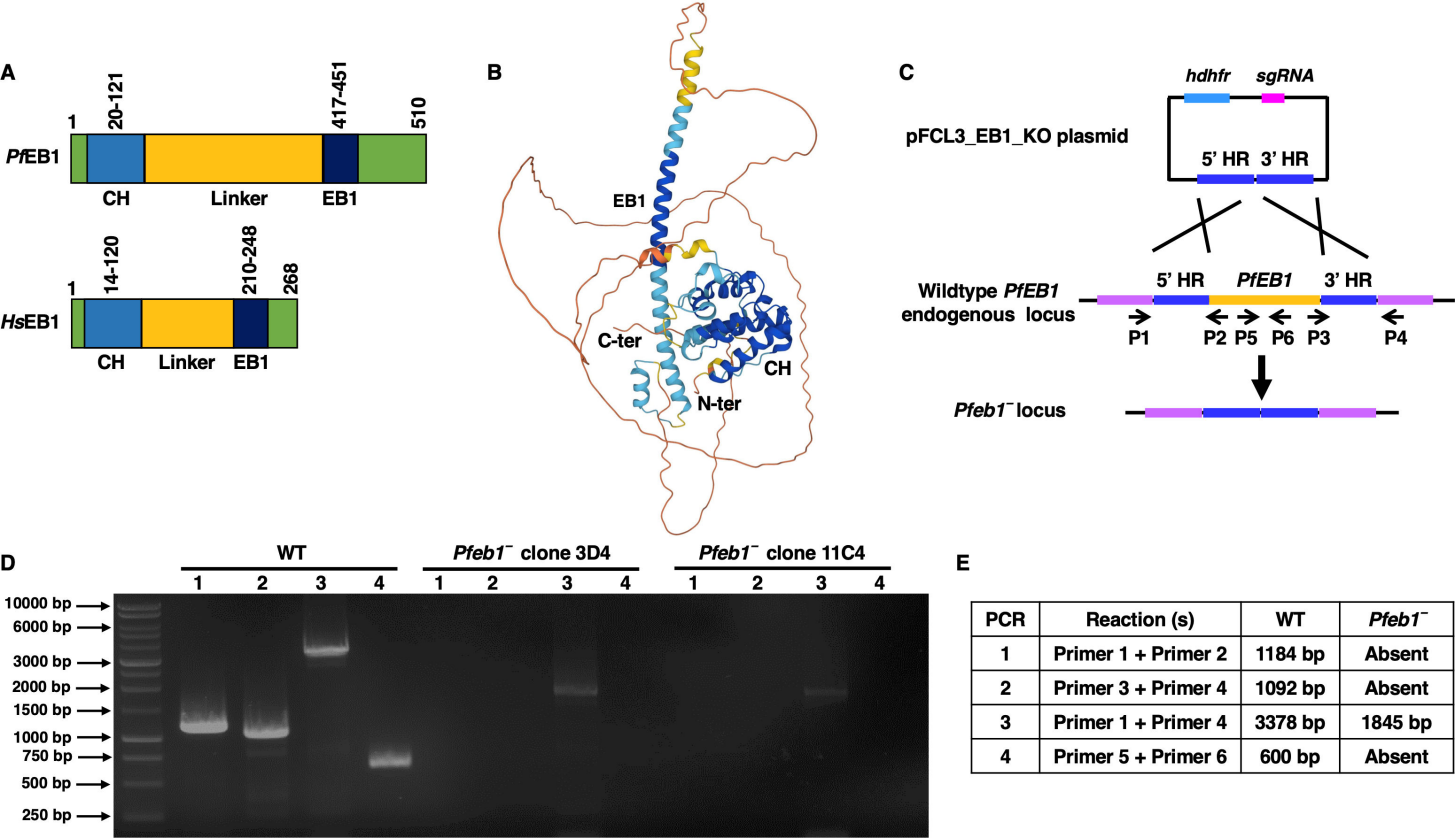
*Pf*EB1 and generation of *Pfeb1^−^* parasites. (**A**) Domain architecture of *Pf*EB1 (upper cartoon) and *Hs*EB1 (lower cartoon) showing the CH domains in blue and the EB1 domains in dark blue. The EB1 domain extends from 417 to 451 amino acids (aa) in *Pf* and from 210 to 248 aa in *Hs*. The linker region is shown in yellow. (**B**) Structural model of *P*fEB1 created using Alphafold (https://alphafold.ebi.ac.uk/). (**C**) The schematic of strategy for *PfEB1* deletion. Oligonucleotides were designed from outside 5′HR and 3′HR. The arrows indicate their positions inside *PfEB1* locus and outside homology arms. (**D**) Confirmation of *Pfeb1^−^* clonal parasites by a set of diagnostic PCRs. (**E**) The expected sizes for different sets of PCRs are shown.

**Fig 2:**
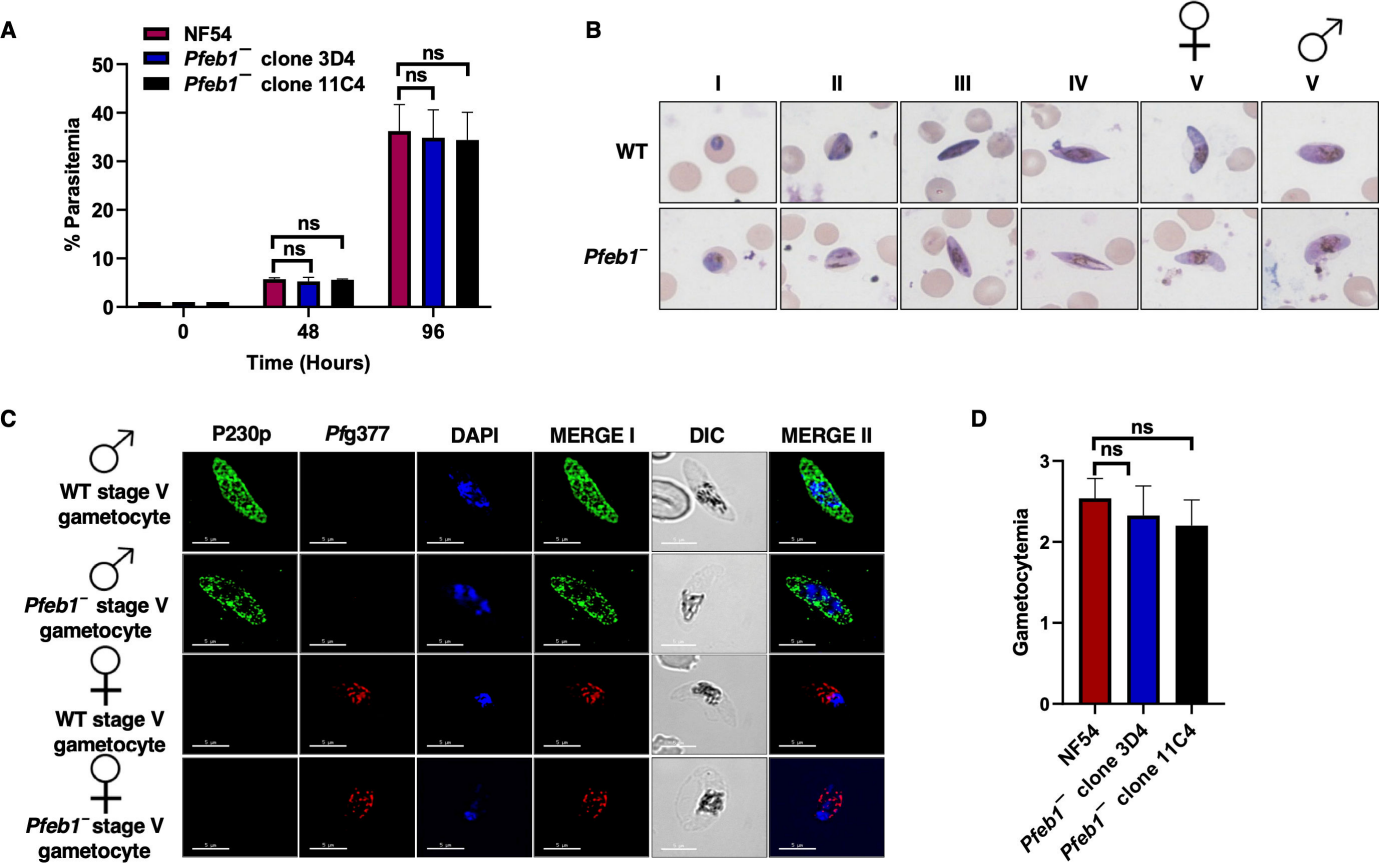
*Pfeb1^−^* asexual stages grow normally and undergo gametocytogenesis. (**A**) Ring-stage synchronous cultures for WT *Pf*NF54 and *Pfeb1^−^* parasites (clones 3D4 and 11C4) were plated, and parasite growth was measured over the course of two asexual replication cycles. Data from three biological replicates were averaged and presented as mean ± standard deviation. ns, not significant. (**B**) Ring-stage synchronous cultures for WT *Pf*NF54 and *Pfeb1^−^* parasites (clones 3D4 and 11C4) were assessed for their ability to form gametocytes. Light microscopy images of Giemsa-stained smears at 100× magnification showed development of WT *Pf*NF54 and *Pfeb1^−^* parasites through the five (I–V) distinct morphological stages. Female and male gametocytes are indicated using sex symbols shown on top of stage V gametocytes. (**C**) Immunofluorescence assays were performed on thin culture smears of mature stage V gametocytes for WT *Pf*NF54 and *Pfeb1^−^* parasites and were stained using anti-*Pf*P230p (green) and anti-*Pf*g377 antisera (red), markers for stage V male and female gametocytes, respectively. Representative images are shown. Parasite DNA was visualized with DAPI (blue). Scale bar = 5 µm. Merge I indicates the merged images for red and green panels. Merge II indicates the merged images for red, green, and blue panels. DAPI, 4′,6-diamidino-2-phenylindole; DIC, differential interest contrast. Male and female gametocytes are indicated using sex symbols shown on the left side of the image panels. (**D**) On day 15 of *in vitro* culture, gametocytemia were measured using thin Giemsa-stained smears. Data from three biological replicates were averaged and presented as mean ± standard deviation. ns, not significant.

*Pf*EB1 is encoded on chromosome 3 with gene identifier PF3D7_0307300 (https://plasmodb.org/plasmo/app/record/gene/PF3D7_0307300). *Pf*EB1 domain analysis using SMART (http://smart.embl-heidelberg.de/) revealed that it contains a calponin homology (CH) domain at the N terminus and a namegiving EB1 domain at the C terminus, separated by a linker region (Fig. 1A). The linker region of *Pf*EB1 is longer than that of Human EB1 (*Hs*EB1) (Fig. 1A). Further structural analysis using Alphafold (https://alphafold.ebi.ac.uk/) revealed a conserved EB1 structure for *Pf*EB1 (Fig. 1B). A sequence alignment of the various *Plasmodium* spp. revealed that EB1 shows high degree of conservation in its CH domain and EB1 domain including a conserved serine residue at amino acid position 15 (S^15^) (Fig. S1). The CH domain of *Hs*EB1 is responsible for the MT binding observed from EB1 localization to MT plus ends (25) and while the EB1 domain binds to adenomatous polyposis coli-binding domain (26, 27). For studying the role of EB1 in parasite development, endogenous *PfEB1* gene deletion was achieved using CRISPR/Cas9 (Fig. 1C). A set of diagnostic PCRs with oligonucleotides specific to the *PfEB1* locus and its upstream (5′) and downstream (3′) regions were used for confirmation of gene deletion parasites (*Pfeb1^−^*) (Fig. 1C to E). Two clones for *Pfeb1^−^* parasites (clones 3D4 and 11C4) were used for phenotypic analysis. *Pfeb1^−^* parasites (clones 3D4 and 11C4) as well as wild-type (WT) NF54 parasites were used for a comparative asexual parasite growth assay. Growth of the parasites was monitored over two continuous asexual replication cycles with Giemsa-stained thin smears prepared from *in vitro* cultures every 48 hours. Microscopic enumeration of parasitemias indicated that *Pfeb1^−^* parasites grow similarly to WT *Pf*NF54 parasites (Fig. 2A), demonstrating that *Pf*EB1 is not critical for asexual blood-stage development and replication.

### *Pfeb1^−^* parasites undergo gametocytogenesis and exhibit normal exflagellation

**Fig 3:**
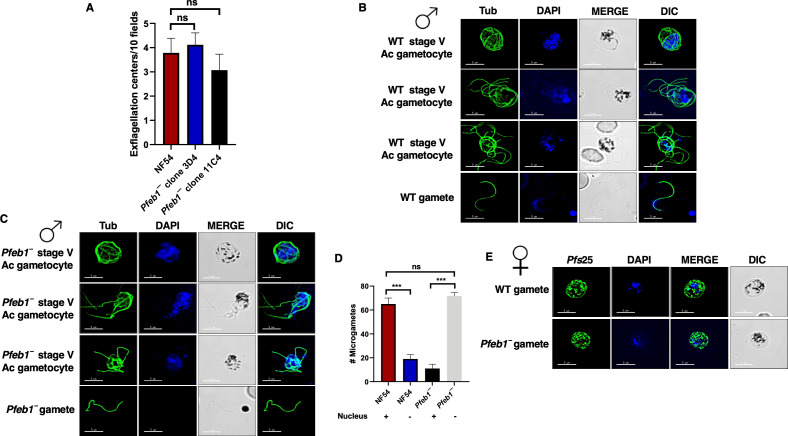
The *Pfeb1^−^* parasites exhibit defect in nuclear segregation during microgametogenesis. (**A**) Exflagellation centers per field were quantified at 15-minute post-activation. Data from three biological replicates were averaged and presented as mean ± standard deviation. *Pfeb1^−^* male gametocytes formed similar exflagellation centers as WT NF54 parasites. (**B**) and (**C**) IFAs were performed on mature stage V microgametocyte thin culture smears. Cultures were activated for 20 minutes *in vitro* for WT *Pf*NF54 or *Pfeb1^−^* (clones 3D4 and 11C4) and stained for α-tubulin (green), a marker for male gametocytes in an IFA. Microgametes emerged from an exflagellating male gametocyte in the WT *Pf*NF54 and *Pfeb1^−^* male gametocytes, shown via α-tubulin staining. (**D**) IFAs were performed on free microgametes for WT *Pf*NF54 and *Pfeb1^−^* using α-tubulin, and parasite DNA was stained with DAPI. Quantitation of microgametes showed normal nuclear segregation for WT NF54 parasites, while the majority of *Pfeb1^−^*-free microgametes did not inherit nuclei. (**F**) IFAs were performed on free macrogametes for WT *Pf*NF54 and *Pfeb1^−^* parasites using α-*Pf*s25, and parasite DNA was stained with DAPI. *Pfeb1^−^* female gametes formed normally like WT NF54 parasites. ns, not significant.

*Pfeb1^−^* parasites were next analyzed for their ability to undergo gametocytogenesis. On day 15 of *in vitro* culture, gametocytemia for both *Pfeb1^−^* parasites (clones 3D4 and 11C4) and WT *Pf*NF54 parasites were scored using Giemsa-stained culture smears and microscopic inspection. *Pfeb1^−^* gametocytemias were similar to WT *Pf*NF54 (Fig. 2D), indicated by their ability to undergo gametocytogenesis, develop through all five gametocyte stages, and further develop into mature male and female gametocytes (Fig. 2B and C). Immunofluorescence assays (IFAs) were performed to better visualize male and female stage V gametocytes using anti-*Pf*P230p (28) and anti-*Pf*g377 antibodies (29), respectively. This revealed normal differentiation of *Pfeb1^−^* parasites into both genders and further confirmed results obtained from Giemsa-stained smears (Fig. 2C). To next analyze the ability of *Pfeb1^−^* mature gametocytes to undergo gametogenesis, day 15 WT *Pf*NF54 and *Pfeb1^−^* parasites were activated by addition of O^+^ human serum and a decrease in temperature from 37°C to room temperature. Wet mounts were prepared using activated gametocyte cultures and then viewed under bright-field microscopic illumination at 40× magnification to measure the exflagellation centers. *Pfeb1^−^* parasites exhibited similar numbers of exflagellation centers as WT parasites, indicating no exflagellation defects (Fig. 3A). We next performed IFAs on activated gametocytes and free microgametes using anti-tubulin antibodies. This revealed that microgamete formation in *Pfeb1^−^* parasites was similar to WT *Pf*NF54 parasites (Fig. 3B and C). During microgametogenesis, male gametocytes rapidly undergo three rounds of DNA replication (8N), which is allocated in eight flagellar microgametes along with axoneme formation. Therefore, we next examined the DNA allocation in free microgametes (Fig. 3B and C). Strikingly, however, examination of free microgametes for WT *Pf*NF54 and *Pfeb1^−^* parasites for DNA staining revealed that the majority of *Pfeb1^−^* microgametes were DAPI negative (Fig. 3D), suggesting a possible male fertility defect. To determine a possible defect in female gametogenesis, we performed IFAs using *Pf*s25 antibodies, which mark activated female gametocytes and macrogametes. These revealed that *Pfeb1^−^* parasites form normal macrogametes like WT *Pf*NF54 parasites (Fig. 3E).

### The *Pfeb1^−^* parasites do not transmit to the mosquito vector due to reduced male fertility

**Fig 4:**
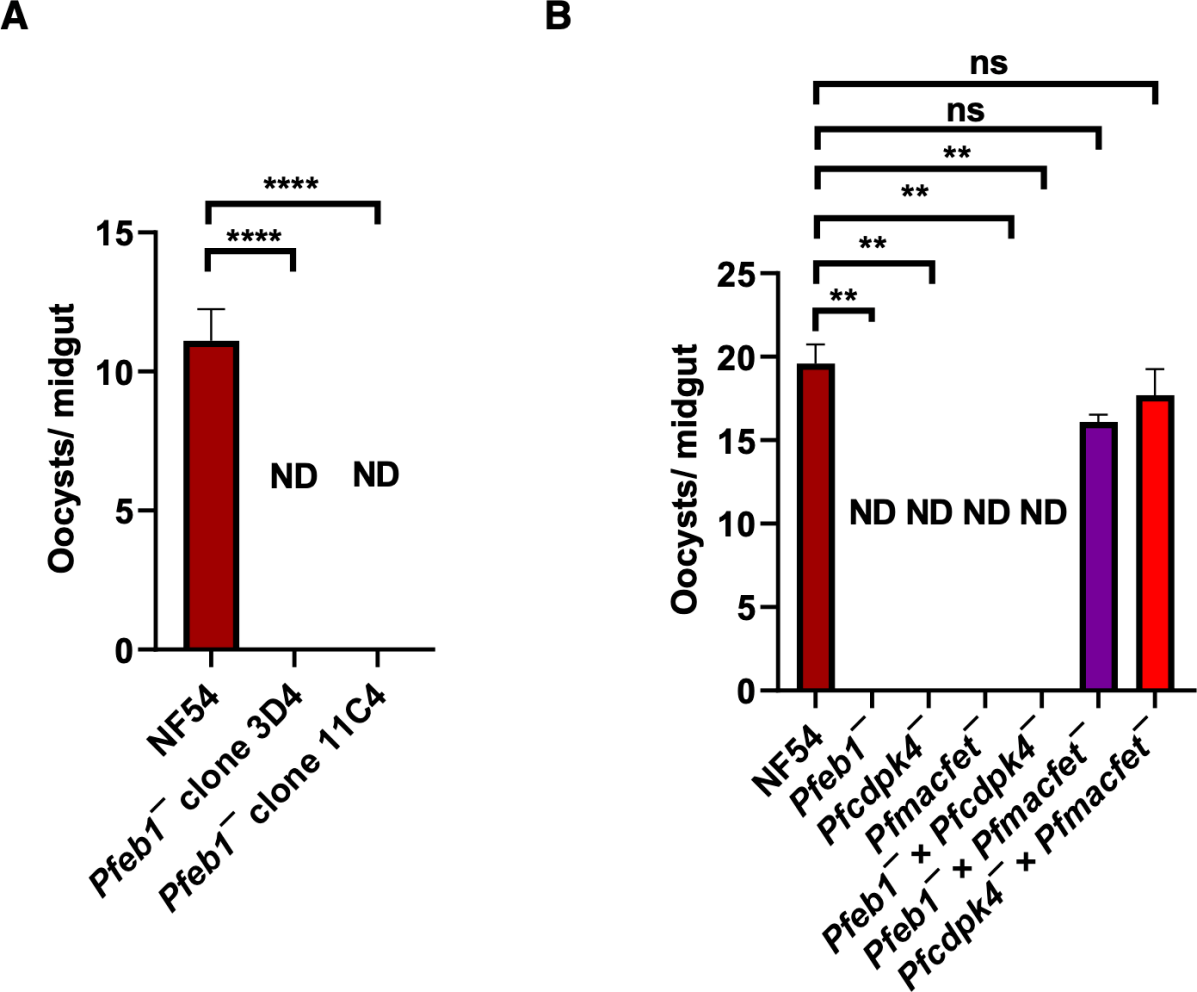
The *Pfeb1^−^* parasites exhibit a robust defect in parasite transmission to the mosquitos. (**A**) On day 7 post-feed, *A. stephensi* mosquitos were dissected, and the number of oocysts per midgut were measured. The *Pfeb1^−^* parasites did not transmit to the mosquitoes. Data from three biological replicates with a minimum of 50 midguts were averaged and presented as mean ± standard deviation. (**B**) On day 7 post-feed, *A. stephensi* mosquitos were dissected, and the number of oocysts per midgut were measured for WT *Pf*NF54, *Pfeb1^−^*, *Pfcdpk4^−^*, *Pfmacfet^−^*, *Pfeb1^−^* × *Pfcdpk4^−^*, *Pfeb1^−^* × *Pfmacfet^−^,* and *Pfcdpk4^−^* × *Pfmacfet^−^. In vitro* genetic crosses revealed a microgamete fertility defect in *Pfeb1^−^* parasites. ND, not detected; ns, not significant.

Next, *Pfeb1^−^* gametocytes were examined for their transmissibility to female *Anopheles stephensi* mosquitoes. Infectious blood meals were prepared for WT *Pf*NF54 and *Pfeb1^−^* stage V gametocytes using standard procedures, and gametocytes were then fed to mosquitoes through membrane feeders. Mosquito midguts, dissected and analyzed under bright-field microscope at 10× magnification on day 7 post-feed, revealed a severe reduction in oocyst numbers for *Pfeb1^−^* parasite infections in comparison to well-infected WT controls (Fig. 4A). Since *P. falciparum* male and female gametocytes exist together, it is not feasible to determine a sex-specific fertility and transmission defect; genetic crosses were performed between *Pfeb1^−^* parasites and male-sterile *Pfcdpk4^−^* parasites (23) and female-sterile *Pfmacfet^−^* parasites (30). For this, the stage V gametocytes from both *Pfeb1^−^* parasites and *Pfcdpk4^−^* parasites or *Pfeb1^−^* parasites and *Pfmacfet^−^* parasites were mixed and fed to the same mosquitoes. The genetic cross between *Pfeb1^−^* parasites and *Pfcdpk4^−^* parasites showed no transmission, while the genetic cross between *Pfeb1^−^* parasites and *Pfmacfet^−^* parasites showed productive transmission. This demonstrated a male-gamete-specific fertility defect in *Pfeb1^−^* gametocytes (Fig. 4B). Taken together, the data show that *Pf*EB1 is critical for parasite transmission to the mosquito vector via a crucial role during microgametogenesis.

## DISCUSSION

To complete the sexual phase of the *P. falciparum* life cycle, a subset of asexually replicating parasites commit to the sexual pathway, followed by differentiation into gametocytes and uptake by *Anopheline* mosquitoes. Environmental triggers within the mosquito midgut activate male gametocyte differentiation into eight flagellated microgametes in a rapid process called exflagellation (13). Microgametes show vigorous motility and, upon encountering a female, initiate fertilization to enable the union of the two haploid genomes and sexual recombination. In *Plasmodium*, the microtubules play important structural roles in the invasive forms (31, 32) as well as in gametocytes, power the motility of microgametes, and play critical role in mitosis (33, 34). During mitosis in *Plasmodium*, the chromosomes are present in an uncondensed state, and the nuclear membrane remains intact as nuclear microtubules capture the kinetochores (34, 35). An electron-dense acentriolar centrosome called the centriolar plaque, which is embedded in a pore in the nuclear envelope, serves as MTOC and is likely present throughout the cell cycle (34, 36, 37). Exflagellation is an astounding cellular process producing eight motile exflagellae, each with a full genome equivalent, from a 1N haploid microgamete following the assembly and stabilization of the nuclei with their associated nuclear envelope non-mitotic MTOC, outer centriolar basal body, and axoneme (38). Proteins involved specifically with basal body function during male gametogenesis of *Plasmodium berghei* (*Pb*) and most likely for *P. falciparum* include SAS6 (39), SAS4 (40), and Kinesin-8B (41). Our study reveals a critical function for nuclear MAP EB1 in nuclear partitioning during *P. falciparum* microgametogenesis, which is crucial for male gamete fertility.

The EB1 family proteins are evolutionarily conserved and have been identified in every organism and nearly all cell types (18). EB proteins comprise of ~300 amino acids with an N-terminal CH domain, a linker region, C-terminal domain, and overlapping coiled-coil and EBH domains, with the CH and coiled-coil domains being conserved in EB1 proteins from diverse organisms (20). However, EB1 is the only member of the +TIP family with an ortholog in *P. falciparum* (12). *Pf*EB1 contains a CH domain and an EB1 domain separated by a linker region. The linker region between the CH and EB1 domains in *P. falciparum* is larger compared to that in *Hs*EB1, despite having similar respective positioning of their EB1 domains and CH domains of similar sizes. *Hs*EB1 is heavily modified at numerous sites (Y^6^, S^7^, S^9^, S^40^, K^60^, K^66^, Y^71^, K^76^, K^83^, K^89^, K^95^, K^100^, Y^119^, K^122^, Y^124^, S^140^, N^147^, K^148^, K^150^, K^151^, T^154^, S^156^, S^155^, S^165^, T^166^, K^174^, K^182^, T^206^, K^212^, Y^217^, K^220^, Y^247^, and Y^268^) (PhosphoSitePlus v6.7.0.1). Notably, K^66^ of *Hs*EB1 is acetylated, ubiquitylated, and crotonylated, with crotonylation of K^66^ functioning in spindle orientation during mitosis (42). So far, no PTMs have been reported for *Pf*EB1, although in our previous data sets, we found S^15^ is phosphorylated in gametocyte stages and hypo-phosphorylated in *Pfcdpk4^−^* parasites, which exhibited a severe male gametogenesis defect (23). The *Pfcdpk4^−^* gametocytes also show hypo-phosphorylation of several parasite kinases, including ARK2, RIO1, and SRPK2 (23). Yeast EB1 (BIM1) is phosphorylated by Aurora kinase homolog lpl1 (43), while *Hs*EB1 co-immunoprecipitates with Aurora kinase B (44). Also, *Hs*EB1 is required to enrich Aurora kinase B at inner centromeres in an MT-dependent manner, resulting in phosphorylation of both kinetochore and other chromatin substrates (45). A recent study in *P. berghei* also demonstrated the interaction between ARK2 and EB1 (46). It is reasonable to propose that *Pf*CDPK4-mediated phosphorylation of ARK2 and/or ARK2-mediated phosphorylation of EB1 at S^15^ may be regulating its microgametogenesis function. This hypothesis is further supported by a recent study demonstrating the role of EB1 S^15^ in spindle-kinetochore attachment during microgametogenesis in *P. yoelii* (47). It will be interesting to explore the role of S^15^ phosphorylation on *Pf*EB1 for its relevance during parasite transmission.

*Pf*EB1 contributes to the functions of an MTOC embedded in the nuclear membrane, which stabilizes and controls the elaborate MT network in gametocytes (12). *Pf*EB1 associates with nuclear MTs, where they bind along the full length of the MT bundles, rather than being strictly localized to the plus end (12). Captured chromatin on the nuclear MT bundles in gametocytes has been indicated by the elongated localization of EB1 in conjunction with the localized regions of chromatin to the nuclear MTs (12). Further heterologous expression experiments have demonstrated that *Py*EB1 has an intrinsic MT-lattice binding property (47). The *Pfeb1^−^* parasites described herein showed that *Pf*EB1 is not required for asexual blood-stage proliferation and gametocytogenesis. Strikingly, the majority of the male *Pfeb1^−^* gametes lack a nucleus, indicating a defect in nuclear division in the absence of EB1, which may result in a motility defect or early death in these microgametes. It is possible that some parasites can compensate for the defects in MT stabilization in the absence of EB1, but there is a delay in mitosis completion, which results in the absence of nuclei in majority of the microgametes. This would be in stark contrast to *Pb*EB1 as *Pbeb1^−^* parasites form normal microgametes and do transmit to the mosquitoes (46). Interestingly, the *Pyeb1^−^* parasites like *Pfeb1^−^* also exhibit a nuclear segregation defect in microgametes which is restored upon complementation with *PfEB1* (47). This reflects a species-specific function for EB1 in different malaria parasites.

Human Kinesin Family Member 18B (KIF18B) is an EB1-binding protein localized to the nucleus during interphase, where it is enriched on astral MT plus ends (48). Furthermore, Kif18B regulates astral MT organization and length during early mitosis (48). Similarly, *P. berghei* kinesin-8B is essential for microgamete formation due to its key role in basal body formation and assembly, as well as structural organization of the axoneme (41). Taken together, kinesin-8B is predicted to interact with EB1 during microgametogenesis. Proper kinetochore assembly is essential for ensuring chromosome segregation during mitosis and, thus, is an essential step in parasite transmission. This process is regulated by centromere proteins CENP-A and CENP-C, kinesins, and MAPs (49). Kinesin-8s, specifically, are associated with MTs and influence MT dynamics (41). The *Plasmodium* genome encodes kinesin-8X and kinesin-8B, the latter of which is essential in microgamete development due to its role in the formation of basal bodies and axoneme development (41). In related Apicomplexan parasite, *T. gondii*, *Tg*EB1 regulates nuclear divisions by controlling spindle dynamics and its association to the kinetochore NDC80 complex (22). Indirectly, *Tg*EB1 secures kinetochore organization and promotes accurate chromosome segregation (22). Both in *P. falciparum* (12) and *P. berghei* (46), EB1 associates with kinetochore marker NDC80 (46), while *Pb*EB1 also associates with basal body marker SAS4 (46). Another basal body marker *Pb*SAS6 is known to have role in nuclear allocation in microgametes (39). Since *Pf*EB1 associates with kinetochore (12) and *Pfeb1^−^* parasites show an abnormal nuclear allocation, we hypothesize that *Pf*EB1 may function in endomitosis during microgametogenesis. The high-resolution microscopy and live-cell imaging experiments would be required to confirm this hypothesis.

In summary, our study demonstrates a crucial role for *Pf*EB1 during male gametogenesis and parasite transmission to the mosquito and links a nuclear microtubule-binding protein to DNA segregation during microgametogenesis.

## MATERIALS AND METHODS

### Reagents and antibodies

The molecular biology reagents were purchased from either MilliporeSigma, USA or Thermo Fisher Scientific, USA, unless otherwise stated. All the restriction enzymes and DNA polymerases were purchased from New England Biolabs, and all the oligonucleotides were purchased from Integrated DNA Technologies, Inc., USA. The following primary antibodies, antisera, and dilutions were utilized: mouse α-tubulin (1:250; Sigma-Aldrich; catalog# T5168), rabbit α-*Pf*g377 (1:250; kindly gifted by Prof. Pietro Alano at Istituto Superiore di Sanità, Rome, Italy) (29), and mouse α-*Pf*P230p (1:200, kindly gifted by Prof. Kim C. Williamson, Uniformed Services University of the Health Sciences, USA (28). Reagents obtained through BEI Resources, NIAID, and NIH include: hybridoma 4B7 α-*Pf*s25-kilodalton gamete surface protein (*Pf*s25), MRA-315, contributed by Louis H. Miller and Allan Saul (1:1 in 3% bovine serum albumin/phosphate-buffered saline, mouse). The Alexa Fluor-conjugated secondary antibodies utilized for IFAs were procured from Thermo Fisher Scientific, USA.

### *P. falciparum* culture and transfection

Standard procedures were followed to culture *P. falciparum* parasites (WT *Pf*NF54 and *Pfeb1^−^*) as asexual blood stages, which received complete RPMI 1640 media, supplemented with 10% (vol/vol) human serum every 24 hours. O^+^ human RBCs (Valley Biomedical, VA, USA) and O^+^ human serum (Valley Biomedical, VA, USA or Interstate Blood Bank, TN, USA) were used to generate *in vitro* gametocytes using methods published elsewhere (50). *PfEB1* Gene deletion parasites, *Pfeb1^−^,* were generated via CRISPR/Cas9 strategy. Recombinant parasites were drug selected, PCR genotyped, and then cloned by limiting dilution. The clonal parasites were confirmed by a set of genotyping PCRs (Fig. 1D and E). Functional assays used two individual clones for *Pfeb1^−^* parasites (clones 3D4 and 11C4).

### Measurement of asexual blood-stage growth and gametocyte development

WT *Pf*NF54 and *Pfeb1^−^* parasites were synchronized at ring stages using 5% sorbitol, and plating was done at equal parasitemia (1%) in six-well plates for comparative analysis of growth rates. Parasite growth for two consecutive asexual replication cycles was quantified by scoring parasitemia on thin Giemsa-stained smears. Likewise, gametocytemia was quantified on day 15 of *in vitro* culture on thin Giemsa-stained smears for comparison of sexual development.

### Indirect immunofluorescence assays

IFAs were performed on asexual and sexual blood-stage parasites and exflagellating microgametocytes using thin smears prepared on Teflon-coated slides as described elsewhere (50). Antigens were visualized using anti-species antibodies. Images were acquired using a 100×  1.4  NA objective 90 (Olympus) on a Delta Vision Elite High-Resolution Microscope (GE Healthcare Life Sciences).

### Exflagellation, standard membrane feeding assay, genetic crosses, and oocyst measurements

Exflagellation, standard membrane feeding assay, and mosquito midgut oocyst measurements for comparative assessments were performed as described elsewhere (51). For performing genetic crosses, *Pfcdpk4^−^*, *Pfmacfet^−^,* and *Pfeb1^−^* parasites were cultured as gametocytes *in vitro*. The day 15 gametocytes from *Pfcdpk4^−^* and *Pfmacfet^−^* were mixed to perform a positive control genetic cross (*Pfcdpk4^−^* × *Pfmacfet^−^*). The day 15 gametocytes for *Pfcdpk4^−^* and *Pfeb1^−^* were mixed to determine a male-specific function (*Pfcdpk4^−^* × *Pfeb1^−^*), while gametocytes for *Pfmacfet^−^* and *Pfeb1^−^* were mixed to determine a female-specific function (*Pfmacfet^−^* × *Pfeb1^−^*) for *Pf*EB1, respectively. These gametocyte mixes were fed to female *A. stephensi* mosquitoes, and day 7 midgut oocysts were enumerated for parasite transmission.

### Statistical analysis

Data collected were expressed as mean ± SD. One-way analysis of variance with post hoc Bonferroni multiple comparison test or unpaired two-tailed Student’s *t* test were used, as indicated, to determine statistical differences. Statistical significances were calculated using GraphPad Prism 9.4, with values of *P* < 0.05 being considered significant. Significance is represented in the figures as follows: ns, not significant, *P* > 0.05; *P < 0.05; **P < 0.01; ***P < 0.001.

## Data Availability

All other relevant data are available from the authors upon reasonable request.

## SUPPLEMENTAL MATERIAL

Fig. S1 mbio.00822-23-s0001.tif The EB1 proteins are highly conserved in *Plasmodium*.

Supplemental text file mbio.00822-23-s0002.docx Supplemental material descriptions.

Table S1 mbio.00822-23-s0003.docx Oligonucleotides used in the study.

ASM does not own the copyrights to Supplemental Material that may be linked to, or accessed through, an article. The authors have granted ASM a non-exclusive, world-wide license to publish the Supplemental Material files. Please contact the corresponding author directly for reuse.
