# Supplementary figures and images for: Plasmodium microtubule-binding protein EB1 is critical for partitioning of nuclei in male gametogenesis
Source: mBio. 2023 Aug 3;14(4):e00822-23. doi: 10.1128/mbio.00822-23 (PMC10470552; doi:10.1128/mbio.00822-23)

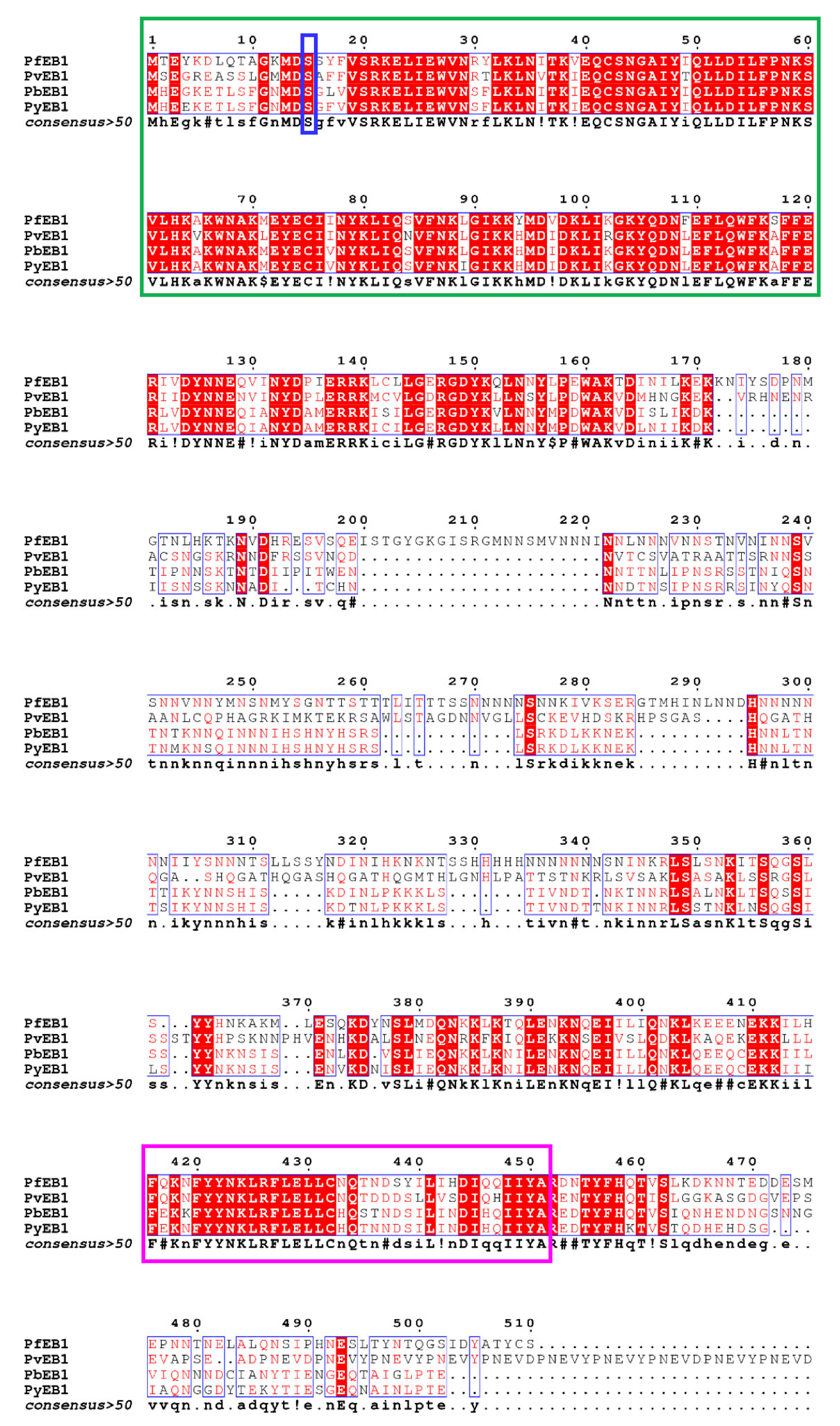

Supplement: Fig. S1 — The EB1 proteins are highly conserved in Plasmodium. [file mbio.00822-23-s0001.tif]
