# Supplementary material for: Plasmodium microtubule-binding protein EB1 is critical for partitioning of nuclei in male gametogenesis
Source: mBio. 2023 Aug 3;14(4):e00822-23. doi: 10.1128/mbio.00822-23 (PMC10470552; doi:10.1128/mbio.00822-23)
Supplement: Supplemental text file — Supplemental material descriptions. [file mbio.00822-23-s0002.docx]

**Supplemental Material includes:**

Supplementary Figure 1 and Supplementary Table S1.

**Supplementary Figure 1. The EB1 proteins are highly conserved in *Plasmodium*.** Sequence alignment for EB1 proteins for *P. falciparum* (*Pf*), *P. vivax* (*Pv*), *P. berghei* (*Pb*) and *P. yoelii* (*Py*). Conserved residues are in white font on a red background. The CH domain is boxed in green, the conserved S^15^ residue is boxed in blue, and the EB1 domain is boxed in pink.

**Supplementary Table S1.** Oligonucleotides used in the study.
