# Supplementary material for: Plasmodium microtubule-binding protein EB1 is critical for partitioning of nuclei in male gametogenesis
Source: mBio. 2023 Aug 3;14(4):e00822-23. doi: 10.1128/mbio.00822-23 (PMC10470552; doi:10.1128/mbio.00822-23)
Supplement: Table S1 — Oligonucleotides used in the study. [file mbio.00822-23-s0003.docx]

**Supplementary Table 1: Oligonucleotides used in the study.**

| **Oligonucleotides used for generation of *Pfeb1¯* parasites**  Oligonucleotides in bold indicate restriction enzyme sites. | |
| --- | --- |
| **Oligo name** | **Forward (5’-3’)** |
| **For amplifying homology arms** | |
| *PfEB1* 5’Homo For | T**GCGGCCGC**GTTTTCAAATAATATATAGCTAAATTTCGAAATAATATTCAAATAGC |
| *PfEB1* 5’Homo Rev | CCAACCCGGGTATAGGCGCGCCTCGAAAAAGAAGATAAGAGAGGAGGAGTAGGAG |
| *PfEB1* 3’Homo For | AGGCGCGCCTATACCCGGGTTGGGAGTAGGAAATGGAAAAATAAAATAAATAAATAAATA |
| *PfEB1* 3’Homo Rev | TAA**GTCGAC**GGTTAACACGTTGTAATATAATGTGTCGTATTAAAATGTGC |
|  | |
| **Guide sequences** | |
| *PfEB1* Guide 1 For | **TATT**AAAGTAGAACAATGTTCTAA |
| *PfEB1* Guide 1 Rev | **AAAC**TTAGAACATTGTTCTACTTT |
| *PfEB1* Guide 2 For | **TATT**GTCCCTTACATATAACACTCA |
| *PfEB1* Guide 2 Rev | **AAAC**TGAGTGTTATATGTAAGGGAC |
|  | |
| ***For Genotyping Pfeb1¯* parasites** | |
| *PfEB1* Geno5 For | GCTTAATTTTCCCTCCCTCCTTTCGACG |
| *PfEB1* Geno5 Rev | CCATTTTGGCATTCCATTTTGCTTTATGCAAAAC |
| *PfEB1* Geno3 For | CATATTAATACATGACATACAACAAATCATATATGCTCGTG |
| *PfEB1* Geno3 Rev | CATTATCTATCATCATTTACCAATCAGACAGTCG |
| *PfEB1 311 For* | TCA**GGATCC**TCTTTATTATCCTCTTACAATGATATAAATATACATAAAAATAAAAATAC |
| *EB1 311 Rev* | ATCC**CTCGAG**TGAACAGTAGGTGGCGTAGTCTATCGA |
